# Structural and Lipidomic Alterations of Striatal Myelin in 16p11.2 Deletion Mouse Model of Autism Spectrum Disorder

**DOI:** 10.3389/fncel.2021.718720

**Published:** 2021-08-12

**Authors:** Jun Ju, Xiuyan Yang, Jian Jiang, Dilong Wang, Yumeng Zhang, Xiaofeng Zhao, Xiaoyi Fang, Huanquan Liao, Lei Zheng, Shupeng Li, Sheng-Tao Hou, Liyang Liang, Yihang Pan, Huiliang Li, Ningning Li

**Affiliations:** ^1^Tomas Lindahl Nobel Laureate Laboratory, The Seventh Affiliated Hospital, Sun Yat-sen University, Shenzhen, China; ^2^Wolfson Institute for Biomedical Research, Division of Medicine, Faculty of Medical Sciences, University College London, London, United Kingdom; ^3^Institute of Developmental and Regenerative Biology, Zhejiang Key Laboratory of Organ Development and Regeneration, Hangzhou Normal University, Hangzhou, China; ^4^Department of Neonatology, The Seventh Affiliated Hospital, Sun Yat-sen University, Shenzhen, China; ^5^The Clinical Neuroscience Center, The Seventh Affiliated Hospital, Sun Yat-sen University, Shenzhen, China; ^6^Department of Anesthesiology, The Seventh Affiliated Hospital, Sun Yat-sen University, Shenzhen, China; ^7^State Key Laboratory of Oncogenomics, School of Chemical Biology and Biotechnology, Peking University Shenzhen Graduate School, Shenzhen, China; ^8^Brain Research Centre and Department of Biology, Southern University of Science and Technology, Shenzhen, China; ^9^Department of Pediatrics, Sun Yat-sen Memorial Hospital, Sun Yat-sen University, Guangzhou, China; ^10^Guangdong Provincial Key Laboratory of Digestive Cancer Research, The Seventh Affiliated Hospital, Sun Yat-sen University, Shenzhen, China; ^11^China-UK Institute for Frontier Science, Shenzhen, China

**Keywords:** autism spectrum disorder, 16p11.2 deletion, striatum, myelin, lipid metabolism

## Abstract

Myelin abnormalities have been observed in autism spectrum disorder (ASD). In this study, we seek to discover myelin-related changes in the striatum, a key brain region responsible for core ASD features, using the 16p11.2 deletion (16p11.2^±^) mouse model of ASD. We found downregulated expression of multiple myelin genes and decreased myelin thickness in the striatum of 16p11.2^±^ mice versus wild type controls. Moreover, given that myelin is the main reservoir of brain lipids and that increasing evidence has linked dysregulation of lipid metabolism to ASD, we performed lipidomic analysis and discovered decreased levels of certain species of sphingomyelin, hexosyl ceramide and their common precursor, ceramide, in 16p11.2^±^ striatum, all of which are major myelin components. We further identified lack of ceramide synthase 2 as the possible reason behind the decrease in these lipid species. Taken together, our data suggest a role for myelin and myelin lipids in ASD development.

## Introduction

Autism spectrum disorder (ASD) is a developmental disorder characterized by social communication difficulties, restricted interests, repetitive behaviors and intellectual disability (Lyall et al., 2017). Signs of ASD generally appear in the first three years of life. The prevalence of ASD is estimated at 0.76% (i.e., 1 in 132 people) globally (Baxter et al., 2015) and around 12.8 per 1000 population in China (Wan et al., 2013). Although the exact cause remains poorly understood, ASD is believed to result from a combination of genetic and environmental factors (Muhle et al., 2004). A recent large-scale genetic analysis of ASD families has identified more than a hundred ASD risk genes, most of which regulate brain development and function (Satterstrom et al., 2020). Copy number variation (CNV), which results in submicroscopic alterations to chromosome structure, represents another genetic risk factor for ASD (Sebat et al., 2007). Notably, CNV involving a ∼600kb DNA segment at the chromosome 16p11.2 locus has been strongly linked to ASD, and 16p11.2 deletion is one of the most frequently observed cytogenetic causes of ASD with an estimated prevalence of 0.5% among ASD patients (Chung et al., 2021).

Interestingly, emerging evidence hints at a link between ASD pathogenesis and abnormal lipid metabolism (Wong et al., 2015; Bjorklund et al., 2020). In the brain, myelin, the multi-layered glial sheath wrapped around neuronal axons, is a major reservoir of brain lipids, with lipids accounting for 70% of myelin dry mass. A number of ASD studies using magnetic resonance imaging (MRI) have implicated brain white matter (mainly made up by myelinated axons) in ASD pathology. For instance, overgrowth of brain white matter was observed in preschool children with ASD (Courchesne et al., 2001), and the severity of ASD symptoms were found to be associated with alterations in white matter development including both overdevelopment and underdevelopment of myelin (Carmody and Lewis, 2010). Moreover, a recent study of autistic adolescents using MRI discovered reduced fibre density in various white matter tracts across the brain (Dimond et al., 2019). In addition, evidence acquired from *Cyfip1* heterozygous rats (an animal model of ASD) has revealed thinning of myelin sheaths in the corpus callosum together with decreased numbers of oligodendrocyte lineage cells including mature oligodendrocytes, the myelin-forming cells (Silva et al., 2019). Taken together, these data imply an important role for myelin and myelin lipids in ASD. So far, how myelin lipid composition changes in ASD remains a mystery.

Here, we investigate myelin-related changes in ASD using the 16p11.2 deletion (16p11.2^±^) mouse line, which is a CNV animal model of ASD with typical autistic phenotypes such as hyperactivity and impaired social ability (Portmann et al., 2014; Wang et al., 2018). Employing this ASD mouse model, we analyze changes in myelin gene expression, myelin structure and lipidomic profile in the striatum, which is a main brain region associated with ASD traits. We present evidence that, compared to wide type (WT) controls, 16p11.2^±^ mice feature decreased expression of myelin genes, reduced levels of key lipid components of myelin, and abnormal myelin microstructure in the striatum. Our data reveal detailed lipidomic changes in the striatum in this ASD animal model, with a focus on alterations to myelin lipid composition.

## Materials and Methods

### Animals

Mice were maintained, with a maximum of 6 mice per cage, in pathogen-free temperature-controlled rooms (22 ± 1°C) under a 12 h light-dark cycle in an animal facility run by the Southern University of Science and Technology (SUST). All animal experiments were performed in accordance with the guidelines for care and use of experimental animals approved by the SUST Animal Care and Use Committee. The 16p11.2^±^ mouse line was imported from the Jackson Laboratory (Stock No: 013128) and maintained in B6/C57 background.

### RNA Sequencing

Female B6/C57 WT and 16p11.2^±^ mice (4 each) were used at postnatal day 60 (P60). Striatum tissues were dissected out from these mice under deep terminal anesthesia and immediately frozen in liquid nitrogen. RNA extraction and RNA Sequencing (RNAseq) analysis were performed by Applied Protein Technology (Shanghai, China). Briefly, total RNA was isolated using TRIzol (Thermo Fisher Scientific, Shanghai) and 2 μg RNA per sample was used as input material to prepare a cDNA library (paired end 250bp) for high-throughput sequencing with the HiSeq 2000 sequencing system (Illumina, Shanghai). After filtering out low quality and adapter sequences, reads were mapped to mouse genome using Hisat2 (Version 2.1.0) (Kim et al., 2015). Read counts were subsequently extracted with the Htseq-count script. DESeq2 (Version 1.28.1) (Love et al., 2014) was used for analyzing differential gene expression. Heatmaps were drawn with TBtools.

### Quantitative Reverse Transcription PCR

Total RNA was extracted from striatum tissues with TRIzol. 3 μg total RNA was reversed-transcribed with random primers, then Quantitative Reverse Transcription PCR (qRT-PCR) was carried out using a SYBR Green Master Mix (Accurate Biology, Changsha, China) and gene specific primers (listed in Supplementary Table 1). Gene expression was normalized against internal control *Gapdh* and the 2^–ΔΔCT^ method was used for gene expression analysis (Livak and Schmittgen, 2001).

### Transmission Electron Microscopy

Mice were transcardially perfused with 4% paraformaldehyde (PFA) in PBS containing 2.5% glutaraldehyde under terminal anesthesia. Striatum tissues were collected and then post-fixed and kept in fresh Transmission Electron Microscopy (TEM) Fixative (Servicebio) at 4°C. The tissues were washed in 0.1 M phosphate buffer (PB, pH 7.4) 3 times, each for 15 min, and then fixed with 1% OsO4 in 0.1 M PB for 2 h at room temperature (RT) in the dark. After washing in 0.1 M PB, the tissues were dehydrated at RT through a series of ethanol (30, 50, 70, 80, 95, and 100% X 2, each for 20 min) and two changes of acetone (each for 15 min). The tissues were embedded in resin, followed by polymerization at 65°C for 48 h. The resin blocks were cut into 60–80 nm thin sections with an ultramicrotome (Leica EM UC7) and the sections were collected onto formvar coated 150 mesh copper grids for staining.

The sections were first stained in 2% alcoholic uranyl acetate for 8 min in the dark, and then after rinsing with 70% ethanol (X 3) and ultra pure water (X 3), they were stained in 2.6% lead citrate for 8 min under CO2 free conditions. After further rinsing with pure water, the sections were dried overnight at RT. Images were taken in a transmission electron microscope and analyzed with Image J for measurement of inner diameters (i.e., axon diameters) and outer diameters of myelin sheaths. Myelin thickness was calculated as the difference between the outer and inner diameters. The myelin g-ratio (the ratio of the inner axonal diameter to outer diameter) was also calculated.

### Lipidomics

Female WT and 16p11.2^±^ mice (6 and 5 respectively) were used at P60 for lipidomic analysis. Striatum tissues were collected from these mice under terminal anesthesia and immediately frozen in liquid nitrogen. Lipid extraction and mass spectrometry (MS)-based lipid detection were performed by Applied Protein Technology. A small portion of each sample in each group was pooled together for use as a quality control sample. Samples were analyzed by liquid chromatography with tandem MS (LC-MS/MS). Lipid identification, peak extraction, peak alignment, and quantification were assessed with LipidSearch 4.1 (Thermo Fisher Scientific) (Taguchi and Ishikawa, 2010).

### Western Blotting

Striatum tissues were lysed in RIPA Buffer (Beyotime, Shanghai) for protein extraction. After centrifugation at 12,000 rpm, 4°C for 15 min, the supernatant was collected and protein concentrations were determined with a PierceTM BCA Protein Assay Kit (Thermo Fisher Scientific). Proteins were separated by SDS-PAGE and transferred to PVDF membranes (Merck Millipore, Guangzhou, China). After blocking with 5% fat-free milk in 1XTBST (0.1% Tween20 in Tris-buffered saline), the blotted membranes were first incubated with primary antibodies (Supplementary Table 2) at 4°C overnight and then with HRP-conjugated secondary antibodies (Supplementary Table 2) at RT for 1 h, with TBST washing in between. Protein immunoreactivity was detected with a Super ECL Detection Kit (Yeasen Biotech, Shanghai), and the signals were visualized with the ChemiDocTM Touch imaging system (Bio-Rad, Shanghai).

### Immunofluorescence

Anesthetized mice were transcardially perfused with 4% PFA. Brain tissues were collected, post-fixed in 4% PFA at 4°C overnight, cryoprotected with 30% sucrose for 2 days, embedded and frozen in Tissue-Tek OCT (Sakura Seiki), and stored at −80°C for cryosectioning. After incubation in blocking buffer (10% goat serum and 0.3% Triton X-100 in PBS), brain sections (30 μm thickness) were first incubated with primary antibodies (Supplementary Table 2) at 4°C overnight and then with second antibodies (Supplementary Table 2) at RT for 1 h, followed by incubation with 1 μg/ml DAPI for 10 min. Slides were mounted with Fluoroshield mounting medium (Beyotime) and imaged with a ZEISS LSM880 confocal microscope.

### Statistics

Unpaired Student’s *t* tests were performed using GraphPad Prism to compare different groups and *p* < 0.05 was considered statistically significant. Data are presented as mean ± SEM.

## Results

### The Expression of Myelin-Related Genes Is Reduced in 16p11.2^±^ Striatum

**FIGURE 1:**
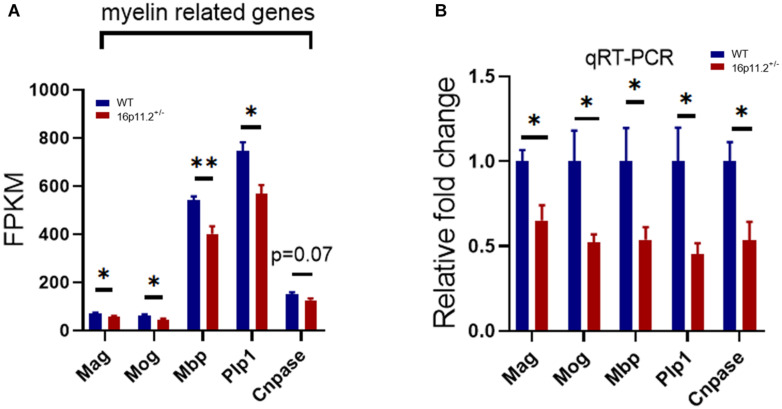
Decreased expression of myelin-related genes in 16p11.2^±^ striatum. **(A)** Decreased expression levels (in FPKM) of myelin-related genes in 16p11.2^±^ striatum. Total RNA was extracted from striatum tissues of 16p11.2^±^ mice (*n* = 4) and WT controls (*n* = 4) at P60 for RNAseq analysis. **(B)** Downregulated mRNA expression (relative to *Gapdh*) of *Mag*, *Mog*, *Mbp*, *Plp1* and *Cnpase* in 16p11.2^±^ striatum as assessed by qRT-PCR (*n* = 4–6 mice). Data were analyzed with unpaired *t* tests (mean ± SEM, *p < 0.05, **p < 0.01). *Mag*, *Myelin associated glycoprotein*; *Mog*, *Myelin oligodendrocyte glycoprotein*; *Mbp*, *Myelin basic protein*; *Plp1*, *Proteolipid protein 1*; *Cnpase*, *Cyclic nucleotide phosphodiesterase*. FPKM, Fragments per kilobase of transcript per million mapped reads.

The striatum is a striped forebrain structure where gray matter bands are separated by white matter tracts of the internal capsule, and is thought potentially responsible for core autism traits. We performed RNAseq to profile gene expression in the striatum of 16p11.2^±^ mice and WT controls at P60. RNAseq data analysis revealed that the expression of multiple myelin-related genes (selected based on a database published by Zhang et al., 2014) was significantly decreased in the striatum of 16p11.2^±^ mice compared to WT controls, including *myelin associated glycoprotein* (*Mag*, *p* < 0.05), *myelin oligodendrocyte glycoprotein* (*Mog*, *p* < 0.05), *myelin basic protein* (*Mbp*, *p* < 0.01), *proteolipid protein 1* (*Plp1*, *p* < 0.05) (Figure 1A). Although not statistically significant, c*yclic nucleotide phosphodiesterase (Cnpase)* expression also showed a sign of reduction (*p* = 0.07) (Figure 1A). In contrast, the expression of genes specific to oligodendrocyte precursor cells (OPCs) including *platelet-derived growth factor receptor a* (*Pdgfra*) and *chondroitin sulfate proteoglycan 4* (*Cspg4*) was unchanged (Supplementary Figure 1). We further confirmed by qRT-PCR that mRNA levels of *Mag*, *Mog*, *Mbp*, *Plp1* and *Cnpase* were indeed reduced (*p* < 0.05) in 16p11.2^±^ striatum (Figure 1B). Taken together, these data indicate downregulated expression of myelin-related genes in the striatum of 16p11.2^±^ mice.

### Myelin Thickness Is Decreased in16p11.2^±^ Striatum

**FIGURE 2:**
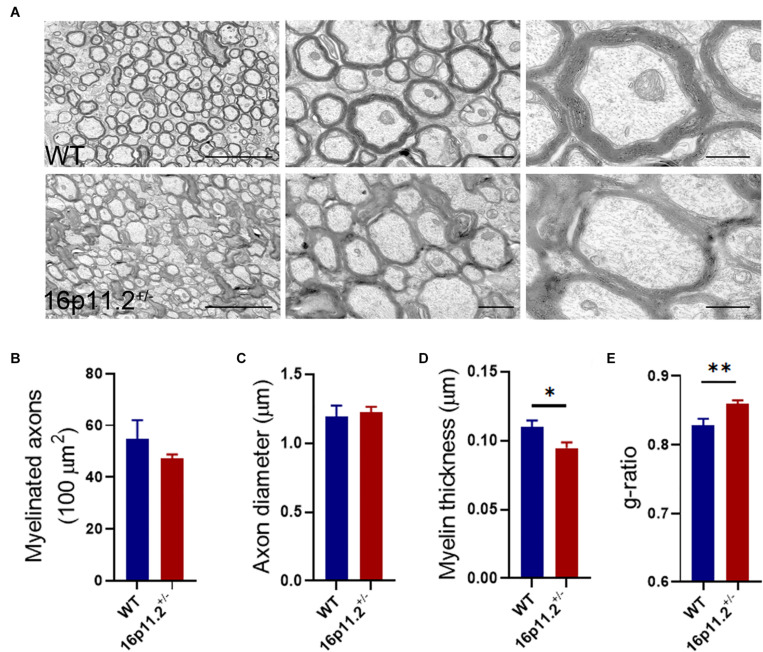
Decreased myelin thickness in 16p11.2^±^ striatum. **(A)** Representative TEM images of myelinated axons in the striatum of P60 16p11.2^±^ mice (lower panels) and WT controls (upper panels). Scale bars, 5 μm (left), 1 μm (middle) and 0.5 μm (right). **(B–E)** Comparison of myelinated axon number **(B)**, axon diameter **(C)**, myelin thickness **(D)**, and myelin g-ratio **(E)** in the striatum of 16p11.2^±^ mice versus WT controls (3 mice per group). All myelinated axons were counted **(B)** but only clearly-defined myelinated axons were measured [**(C–E)**, WT: *n* = 267, 16p11.2^±^: *n* = 131] in 15 microscope fields (5 field per mouse) under study for each group. Data were analyzed by unpaired *t* tests (mean ± SEM, *p < 0.05, **p < 0.01).

To find out myelin structural changes in16p11.2^±^ striatum, we preformed TEM with striatum tissues from P60 16p11.2^±^ mice and WT controls (3 mice per group; Figure 2). We counted all myelinated axons in 15 microscope fields (5 fields per mouse) under study for each group and found no significant difference in the number of myelinated axons between the two groups (16p11.2^±^ vs WT: 47.3 ± 1.5 vs 54.7 ± 7.4 per 100 μm^2^; Figure 2B). In addition, only clearly-defined myelinated axons were measured, 131 and 267 in total for 16p11.2^±^ mice and WT controls respectively. Dysmyelinated axons with abnormal morphology, which were often encountered in 16p 11.2^±^ striatum, were excluded from measurement to avoid inaccuracy. Although there was no appreciable change in axon diameter in the striatum of 16p11.2^±^ mice relative to WT controls (16p11.2^±^ vs WT: 1.224 ± 0.041 μm vs 1.192 ± 0.081 μm), decreased thickness of myelin sheaths (16p11.2^±^ vs WT: 0.095 ± 0.004 μm vs 0.11 ± 0.005 μm; *p* < 0.05) along with a higher g-ratio (16p11.2^±^ vs WT: 0.859 ± 0.005 vs 0.829 ± 0.009; *p* < 0.01) was observed in 16p11.2^±^ striatum (Figure 2). Together, these results reveal altered myelin microstructure and thinning of myelin sheaths in 16p11.2^±^ striatum.

### Oligodendrocyte Lineage Cell Numbers Are Unchanged in 16p11.2^±^ Striatum

**FIGURE 3:**
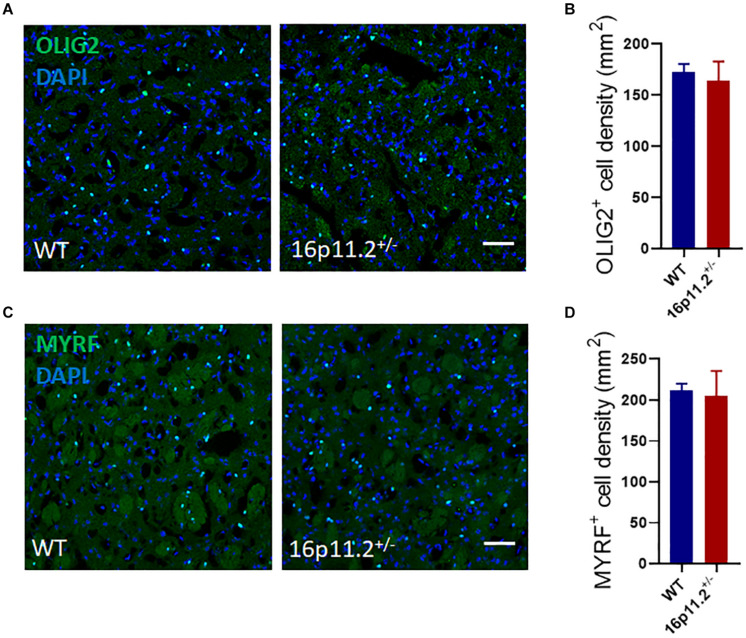
Oligodendrocyte lineage cell numbers are unchanged in 16p11.2^±^ striatum. **(A,C)** Representative images of OLIG2^+^ [green, **(A)**] oligodendrocyte lineage cells and MYRF^+^ [green, **(C)**] differentiated/mature oligodendrocytes in striatum sections of P60 16p11.2^±^ mice and WT controls. Nuclei were counterstained with DAPI (blue). **(B,D)** Comparison of OLIG2^+^**(B)** and MYRF^+^ **(D)** cell numbers in the striatum of 16p11.2^±^ mice versus WT controls, showing no significant differences (data from 4 mice per group, mean ± SEM, unpaired *t* test). Scale bar = 50 μm.

To explore the reason behind the reduced expression of myelin genes, we examined the numbers of oligodendrocyte lineage cells in the striatum of P60 16p11.2^±^ and WT mice by immunostaining. No difference was found in the number of either OLIG2^+^ oligodendrocyte lineage cells or MYRF^+^ differentiated/mature oligodendrocytes between 16p11.2^±^ and WT striatum (Figure 3). In addition, 16p11.2^±^ mice also exhibited normal numbers of OLIG2^+^ and MYRF^+^ cells in the striatum at P14, suggesting that early postnatal OL generation is unaffected in 16p11.2^±^ mice (Supplementary Figure 2).

### Lipidomic Profile Is Altered in 16p11.2^±^ Striatum

**FIGURE 4:**
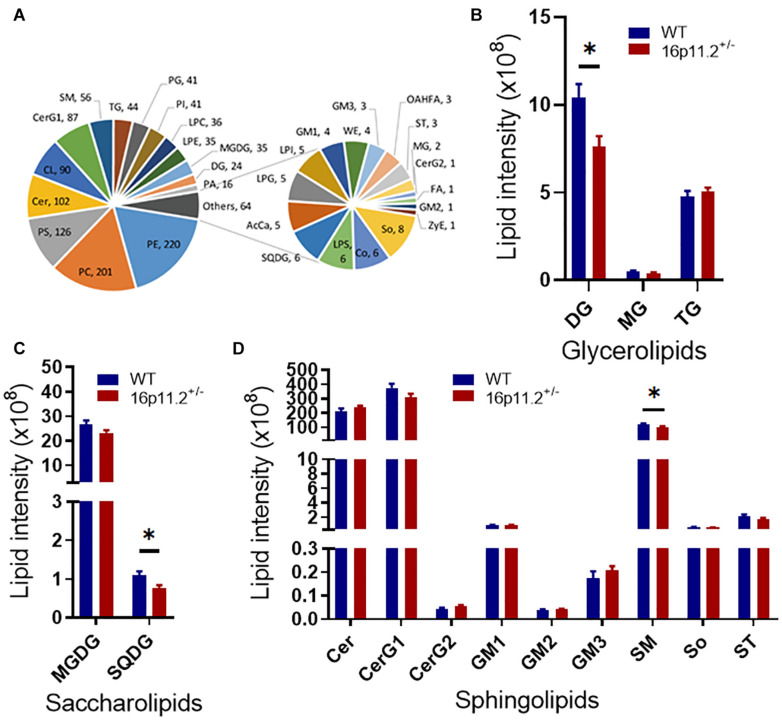
Altered lipidomic profile in 16p11.2^±^ striatum. Lipidomics analysis was performed with striatum tissues from P60 16p11.2^±^ mice (*n* = 5) and WT controls (*n* = 6). A total of 32 lipid classes and 1218 lipid species were detected. **(A)** Charts illustrating all detected lipid classes. Each tail number indicates the number of lipid species detected. **(B–D)** Comparison of lipid classes of glycerolipids **(B)**, saccharolipids **(C)**, and sphingolipids **(D)** in 16p11.2^±^ mice versus WT controls. Data were analyzed with unpaired *t* tests (mean ± SEM, *p < 0.05). Cer, ceramide; CerG1, hexosyl ceramide; CerG2, dihexosyl ceramide; DG, diglyceride; GM1, gangliosides 1; GM2, gangliosides 2; GM3, gangliosides 3; MG, monoglyceride; MGDG, monogalactosyldiacylglycerol; SM, sphingomyelin; So, sphingosine; SQDG, sulfoquinovosyldiacylglycerol; ST, sulfatide; TG, triglyceride.

Given the high proportion of lipid (70–85%) in the makeup of myelin and the linkage between dysregulation of lipid metabolism and ASD (El-Ansary et al., 2020; Montani, 2021), we asked how myelin lipid composition was affected in the striatum of 16p11.2^±^ mice. We performed lipidomics analysis of striatum tissues from P60 16p11.2^±^ and WT mice using LC-MS/MS. A total of 1218 lipid species from 32 lipid classes were detected, belonging to 7 lipid families - glycerolipids, saccharolipids, sphingolipids, fatty acyls, glyceropholipids, sterol lipids and prenol lipids (Figure 4A). Notably, three lipid classes were significantly reduced in the striatum of 16p11.2^±^ mice compared to WT controls (*p* < 0.05), including diglyceride (DG, glycerolipid; Figure 4B), sulfoquinovosyldiacylglycerol (SQDG, saccharolipid; Figure 4C), and sphingomyelin (SM, sphingolipid), a major myelin lipid class (Figure 4D). In addition, hexosyl ceramide (CerG1, sphingolipid), another main myelin lipid class, displayed a tendency to decrease in 16p11.2^±^ striatum (Figure 4D). No differences were observed in the levels of fatty acyls, glycerophospholipids, sterol lipids and prenol lipids between 16p11.2^±^ and WT mice (Supplementary Figure 3).

### SM and CerG1 Species Are Decreased in 16p11.2^±^ Striatum

**FIGURE 5:**
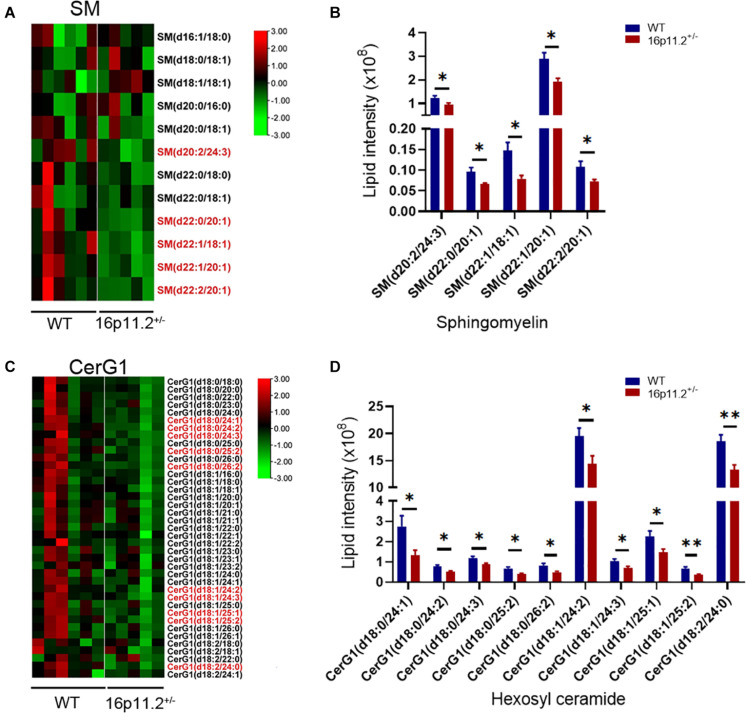
Decreased SM and CerG1 species in 16p11.2^±^ striatum. **(A,C)** Heatmaps of SM **(A)** and CerG1 **(C)** species identified in the striatum of P60 16p11.2^±^ mice (*n* = 5) and WT controls (*n* = 6) by lipidomics analysis. The lipid species showing a significant change in level in 16p11.2^±^ striatum are written in red. **(B,D)** Charts of comparing the “red” SM **(B)** and CerG1 **(D)** species, indicating their decreased levels in the striatum of 16p11.2^±^ mice relative to WT controls (mean ± SEM, *p < 0.05/**p < 0.01 for unpaired *t* tests).

SM and CerG1 are both sphingolipid, comprising a fatty acid (FA) chain joined to a long chain sphingoid base (i.e., C18 sphingosine or a shorter/longer variant) via an amide linkage (Supplementary Figure 4). Given that SMs and CerG1s are pivotal components of myelin, we evaluated the levels of SM and CerG1 species in 16p11.2^±^ mice versus WT controls using our lipidomics data and identified the SM and CerG1 species showing significantly changed levels (all decreased) in 16p11.2^±^ striatum (Figure 5). A drop (*p* < 0.05) was observed in the levels of several long acyl-chain SM species in 16p11.2^±^ striatum, including SM(d20:2/24:3), SM(d22:0/20:1), SM(d22:1/18:1), SM(d22:1/20:1), and SM(d22:2/20:1) (Figures 5A,B). Similarly, a decrease (*p* < 0.05/*p* < 0.01) was found in the levels of 10 CerG1 species (all having a very long/C24-C26 FA chain) in 16p11.2^±^ striatum, including CerG1(d18:0/24:1), CerG1(d18:0/24:2), CerG1(d18:0/24:3), CerG1(d18:0/25:2), CerG1(d18:0/26:2), CerG1(d18:1/24:2), CerG1(d18:1/24:3), CerG1(d18:1/25:1), CerG1(d18:1/25:2), and CerG1(d18:2/24:0) (Figures 5C,D).

### Ceramide (Cer) Species Are Decreased in 16p11.2^±^ Striatum

**FIGURE 6:**
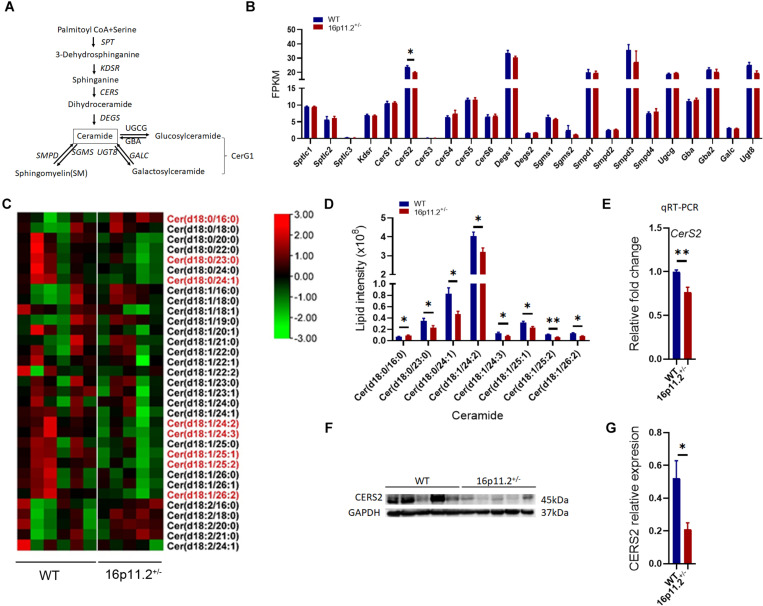
Decreased Cer species and *CerS2* expression in 16p11.2^±^ striatum. **(A)** Diagram of ceramide metabolism. **(B)** Expression levels of genes involved in ceramide synthesis revealed by RNAseq analysis using RNA isolated from striatum tissues of P60 16p11.2^±^ mice (*n* = 4) and WT controls (*n* = 4). Only *CerS2* expression was significantly reduced in 16p11.2^±^ striatum. **(C)** Heatmaps of Cer species identified by lipidomics analysis in the striatum of P60 16p11.2^±^ mice (*n* = 5) and WT controls (*n* = 6). The Cer species displaying a significant change in level in 16p11.2^±^striatum are written in red. **(D)** Chart of comparing the “red” Cer species, indicating their decreased levels in the striatum of 16p11.2^±^ mice in relation to WT controls. **(E)** Reduced *CerS2* expression (relative to *Gapdh*) in 16p11.2^±^ striatum as assessed by qRT-PCR (*n* = 4 mice). **(F,G)** Reduced CERS2 expression in 16p11.2^±^ striatum revealed by Western blotting (*n* = 5 mice). CERS2 expression was examined by Western blotting **(F)** and normalized against GAPDH for quantitative comparison. **(G)** Data were analyzed with unpaired *t* tests (mean ± SEM, *p < 0.05, **p < 0.01). FPKM, Fragments per kilobase of transcript per million mapped reads.

Echoing the decrease in SM and CerG1 species, our lipidomics analysis also revealed reduced levels (*p* < 0.05/*p* < 0.01) of certain species of Cer (the common precursor of SM and CerG1) in 16p11.2^±^ striatum, including Cer(d18:0/23:0), Cer(d18:0/24:1), Cer(d18:1/24:2), Cer(d18:1/24:3) Cer(d18:1/25:1), Cer(d18:1/25:2) and Cer(d18:1/26:2) (Figures 6C,D). Cer provides a lipid backbone for all complex sphingolipids. Given the vital role of Cer in the synthesis of SM and CerG1, we analyzed the expression of genes involved in Cer metabolism (Figure 6A) employing our RNAseq data. Only *ceramide synthase 2* (*CerS2*) expression was significantly lower (*p* < 0.05) in the striatum of 16p11.2^±^ mice than in WT controls (Figure 6B), whereas the other relevant genes including *CerS1* and *CerS3-6* exhibited no significant changes in mRNA levels in 16p11.2^±^ striatum (Figure 6B).

In Cer biosynthesis, the addition of a FA chain to a sphingoid base is catalyzed by CERS1-6 (Figure 6A). Each CERS displays a specificity to the FA chain length. CERS2 and CERS4 preferentially use C20-C26 and C18-C20 FA residues respectively, whereas CerS5, 6 prefer shorter C14-C16 FA chains, and CERS1 shows high specificity to C18 FA residues (Levy and Futerman, 2010). To verify data from RNAseq analysis, we conducted qRT-PCR to assess *CerS2* expression in the striatum of P60 16p11.2^±^ mice versus WT controls and confirmed that *CerS2* expression was markedly decreased (*p* < 0.01) in 16p11.2^±^ striatum (Figure 6E), which was further validated at protein level (*p* < 0.05) by Western blotting (Figures 6F,G). Taken together, these data suggest that lack of CERS2 could be the reason behind the decreased Cer, SM and CerG1 species in 16p11.2^±^ striatum.

## Discussion

Myelin abnormalities have been implicated in a variety of neurological disorders including ASD (Galvez-Contreras et al., 2020). Evidence from both ASD patients and ASD animal models have revealed structural changes in brain white matter (Courchesne et al., 2001; Carmody and Lewis, 2010; Dimond et al., 2019; Silva et al., 2019). In this study, we found downregulated expression of a number of myelin genes (Figure 1) and altered myelin microstructure (Figure 2) in the striatum of 16p11.2^±^ mice, a CNV animal model of ASD. The striatum, a striated structure of gray/white matter in the brain, is possibly a key contributor to core ASD features (Peca et al., 2011). In fact, abnormalities of striatal myelin/myelination have already been reported in ASD animal models. For instance, in the *fragile X mental retardation 1* (*Fmr1*) knockout mouse model of ASD, MRI scans pointed to a reduction in myelin macromolecular content (i.e., proteins and lipids) in the internal capsule as well as other white matter regions at P60 (Shi et al., 2019). In addition, in *chromodomain-helicase-DNA-binding protein 8* (*Chd8*) conditional knockout mice where *Chd8* is specifically ablated in oligodendrocyte lineage cells, autism-like behavioral changes appeared to correlate with myelin microstructural changes in the striatum (Kawamura et al., 2020). It has been suggested that *Fmr1 or Chd8* knockout can lead to decreased numbers of oligodendrocyte lineage cells and delayed myelination in brain development, but in 16p11.2^±^ mice, we did not observe any significant changes in the numbers of oligodendrocyte lineage cells in the striatum at P60 (Figure 3). These differing observations may result from different molecular mechanisms at play in different ASD models, reflecting the genetic complexity of ASD. Nonetheless, myelin impairment has proved to play a part in ASD development.

Myelin is the predominant host of many lipid classes in the brain. Currently, little is known about how brain lipid composition changes in ASD. Several lipidomics studies have been conducted in ASD patients, most of which used peripheral blood samples (Ventura et al., 2020). Notably, a recent study uncovered age-related changes in lipidomic composition in the gray matter of human prefrontal cortex and attributed increased levels of Cers and other sphingolipids detected in this gray matter region in ASD patients (age 18–65) more to ASD than to age or postmortem interval (Yu et al., 2020). Our study reveals here lipidomic changes in the myelin-rich striatum in detail in the 16p11.2^±^ ASD mouse model, highlighting decreased levels of key lipid components of myelin - CerG1, SM and Cer species - in 16p11.2^±^ striatum (Figures 4–6). Interestingly, in the maternal immune activation (MIA) mouse model of ASD, where autism-like symptoms arise from acute hyperpurinergia, the plasma levels of 15 Cer and 15 SM species (most having a long acyl chain) have been shown to be significantly reduced (Zolkipli-Cunningham et al., 2021). Taken together, a commonality between these observations regarding ASD is altered lipid metabolism. Given the lipid-rich nature of myelin, it is possible that alterations to myelin lipid composition (i.e., myelin abnormalities) could be a common driving factor in ASD development. In addition, it would be worth exploring whether altered levels of myelin lipid species are indicative of changes in plasma levels of lipid species in ASD, with a view to finding new biomarkers for ASD.

We further identified CERS2 as being responsible for the reduced levels of sphingolipids in 16p11.2^±^ striatum (Figure 6). The range for substrate specificity of CERS2 is C20-C26 FA residues, though CERS2 prefers longer FA chains. Therefore, the downregulation of CERS2 expression seems able to explain the decreased levels of long/very long acyl-chain (C18-C26) species of Cer (C23-C26), CerG1 (C24-C26) and SM (C18-C24) in 16p11.2^±^ striatum. It has been reported that lack of CERS2 leads to a steady decline in myelin integrity together with loss of MBP in *CerS2* knockout mice (Imgrund et al., 2009), though it is not clear if CERS2 deficiency is the direct cause of reduced myelin gene expression. To evaluate CERS2’s potential as a therapeutic target would require us to first find out if downregulation of CERS2 also occurs in other ASD models and if CERS2 overexpression could alleviate myelin defects in these ASD models.

In addition, *family with sequence similarity 57, member b* (*Fam57b*) gene, located in the deleted DNA region of 16p11.2^±^ mice, encodes a protein containing a TLC domain that is shared by CERS (Yamashita-Sugahara et al., 2013). In our RNAseq data, *Fam57b* expression was found near background level in the striatum of both 16p11.2^±^ and WT mice, and it was below detection limit when assessed by qRT-PCR (data not shown). Therefore, the possibility of Fam57b haploinsufficiency having an impact on Cer, CerG1 and SM levels in 16p11.2^±^ striatum can be ruled out.

## Data Availability Statement

The data generated in this manuscript can be found here https://www.ncbi.nlm.nih.gov/geo/query/acc.cgi?acc=GSE179056.

## Ethics Statement

The animal study was reviewed and approved by Southern University of Science and Technology Animal Care and Use Committee.

## Author Contributions

NL, HLi, and YP designed the project, guided the project, and revised the manuscript. JJu, XY, and JJi did the experiments and wrote the draft. DW, YZ, XZ, XF, HLia, LZ, SL, S-TH, and LL helped to design the project, guided the project all through, and revised the manuscript. All authors contributed to the article and approved the submitted version.

## Conflict of Interest

The authors declare that the research was conducted in the absence of any commercial or financial relationships that could be construed as a potential conflict of interest.

## Publisher’s Note

All claims expressed in this article are solely those of the authors and do not necessarily represent those of their affiliated organizations, or those of the publisher, the editors and the reviewers. Any product that may be evaluated in this article, or claim that may be made by its manufacturer, is not guaranteed or endorsed by the publisher.

## Supplementary Material

Supplementary Figure 1 Unchanged expression of OPC-specific genes in 16p11.2^±^ striatum. No differences were found in the expression levels (in FPKM) of OPC-specific genes *Pdgfra* and *Cspg4* in the striatum between P60 16p11.2^±^ mice (*n* = 4) and WT controls (*n* = 4) by RNAseq analysis (mean ± SEM, unpaired *t* test). FPKM: Fragments per kilobase of transcript per million mapped reads.

Supplementary Figure 2 Oligodendrocyte lineage cell numbers are unchanged in 16p11.2^±^ striatum at P14. **(A,C)** Representative images of OLIG2^+^ [green, **(A)**] oligodendrocyte lineage cells and MYRF^+^ [green, **(C)**] differentiated/mature oligodendrocytes in striatum sections of 16p11.2^±^ mice and WT controls at P14. **(B,D)** Comparison of OLIG2 ^+^ **(B)** and MYRF^+^ **(D)** cell numbers in 16p11.2^±^ mice versus WT controls, showing no significant differences (data from 4 mice per group, mean ± SEM, unpaired *t* test). Scale bar = 50 μm.

Supplementary Figure 3 The lipid classes showing unchanged levels in 16p11.2^±^ striatum. **(A–D)** No differences were found in the levels of detected classes of fatty acyls **(A)**, sterol lipids **(B)**, prenol lipids **(C)** and glycerophospholipids **(D)** in the striatum between 16p11.2^±^ mice (*n* = 5) and WT controls (*n* = 6) at P60 by lipidomics analysis (mean ± SEM, unpaired *t* test). AcCa, acyl Carnitine; CL, cardiolipin; Co, coenzyme; FA, fatty acid; LPC, lysophosphatidylcholine; LPE, lysophosphatidylethanolamine; LPG, lysophosphatidylglycerol; LPI, lysophosphatidylinositol; LPS, lysophosphatidylserine; OAHFA, (O-acyl)-1-hydroxy fatty acid; PA, phosphatidic acid; PC, phosphatidylcholine; PE, phosphatidylethanolamine; PG, phosphatidylglycerol; PI, phosphatidylinositol; PS, phosphatidylserine; WE, wax exters; ZyE, zymosterol.

Supplementary Figure 4 The structural formula of sphinosine, ceramide, sphingomyelin and hexosyl ceramide drawn with ChemBioDraw Ultra 14.0. **(A)** Sphingosine with a C18 chain. **(B)** Ceramide, comprising a sphingosine backbone amide-linked to a fatty acid chain. **(C)** Sphingomyelin, formed by ceramide linking up with a phosphocholine head group. **(D)** Hexosyl ceramide, composed of a ceramide linked to a hexose.

Supplementary Table 1 List of primers used for qRT-PCR.

Supplementary Table 2 List of primary antibodies; List of secondary antibodies.
