# Supplementary figures and images for: Structural and Lipidomic Alterations of Striatal Myelin in 16p11.2 Deletion Mouse Model of Autism Spectrum Disorder
Source: Front Cell Neurosci. 2021 Aug 12;15:718720. doi: 10.3389/fncel.2021.718720 (PMC8416256; doi:10.3389/fncel.2021.718720)

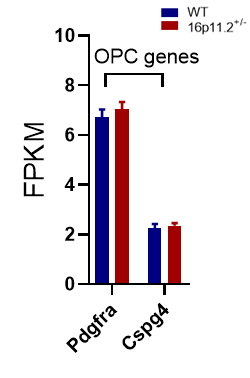

Supplement: Supplementary Figure 1 — Unchanged expression of OPC-specific genes in 16p11.2± striatum. No differences were found in the expression levels (in FPKM) of OPC-specific genes Pdgfra and Cspg4 in the striatum between P60 16p11.2± mice (n = 4) and WT controls (n = 4) by RNAseq analysis (mean ± SEM, unpaired t test). FPKM: Fragments per kilobase of transcript per million mapped reads. [file Image_1.TIF]

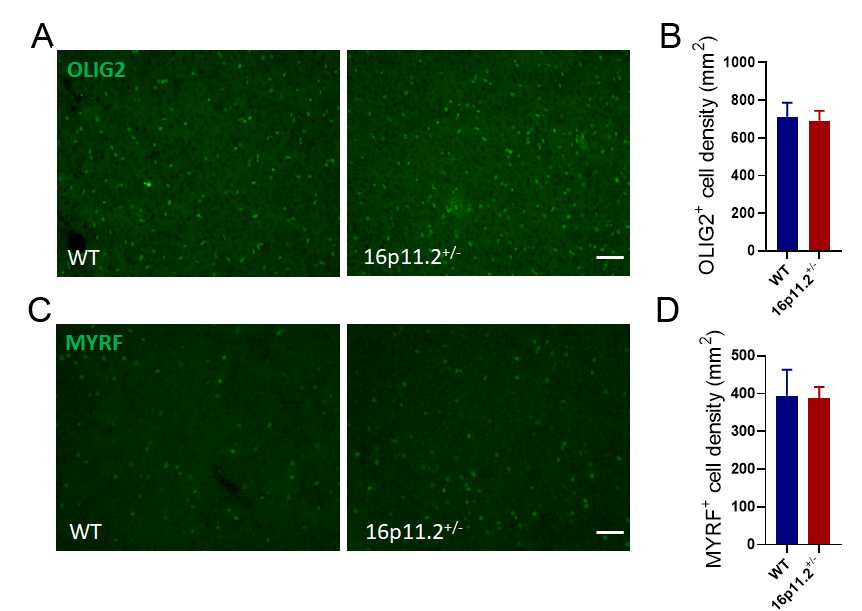

Supplement: Supplementary Figure 2 — Oligodendrocyte lineage cell numbers are unchanged in 16p11.2± striatum at P14. (A,C) Representative images of OLIG2+ [green, (A)] oligodendrocyte lineage cells and MYRF+ [green, (C)] differentiated/mature oligodendrocytes in striatum sections of 16p11.2± mice and WT controls at P14. (B,D) Comparison of OLIG2 + (B) and MYRF+ (D) cell numbers in 16p11.2± mice versus WT controls, showing no significant differences (data from 4 mice per group, mean ± SEM, unpaired t test). Scale bar = 50 μm. [file Image_2.TIF]

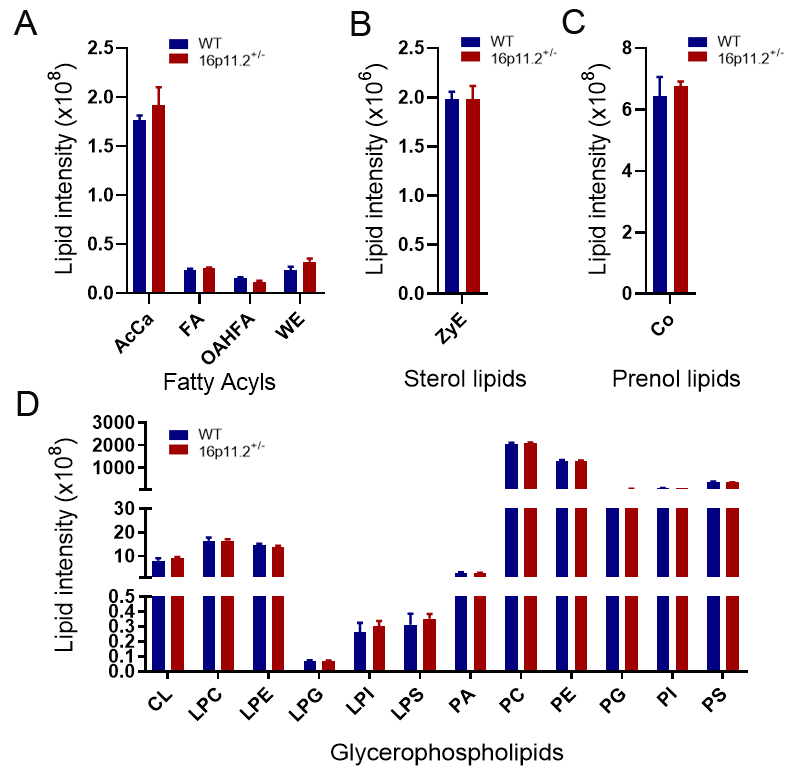

Supplement: Supplementary Figure 3 — The lipid classes showing unchanged levels in 16p11.2± striatum. (A–D) No differences were found in the levels of detected classes of fatty acyls (A), sterol lipids (B), prenol lipids (C) and glycerophospholipids (D) in the striatum between 16p11.2± mice (n = 5) and WT controls (n = 6) at P60 by lipidomics analysis (mean ± SEM, unpaired t test). AcCa, acyl Carnitine; CL, cardiolipin; Co, coenzyme; FA, fatty acid; LPC, lysophosphatidylcholine; LPE, lysophosphatidylethanolamine; LPG, lysophosphatidylglycerol; LPI, lysophosphatidylinositol; LPS, lysophosphatidylserine; OAHFA, (O-acyl)-1-hydroxy fatty acid; PA, phosphatidic acid; PC, phosphatidylcholine; PE, phosphatidylethanolamine; PG, phosphatidylglycerol; PI, phosphatidylinositol; PS, phosphatidylserine; WE, wax exters; ZyE, zymosterol. [file Image_3.TIF]

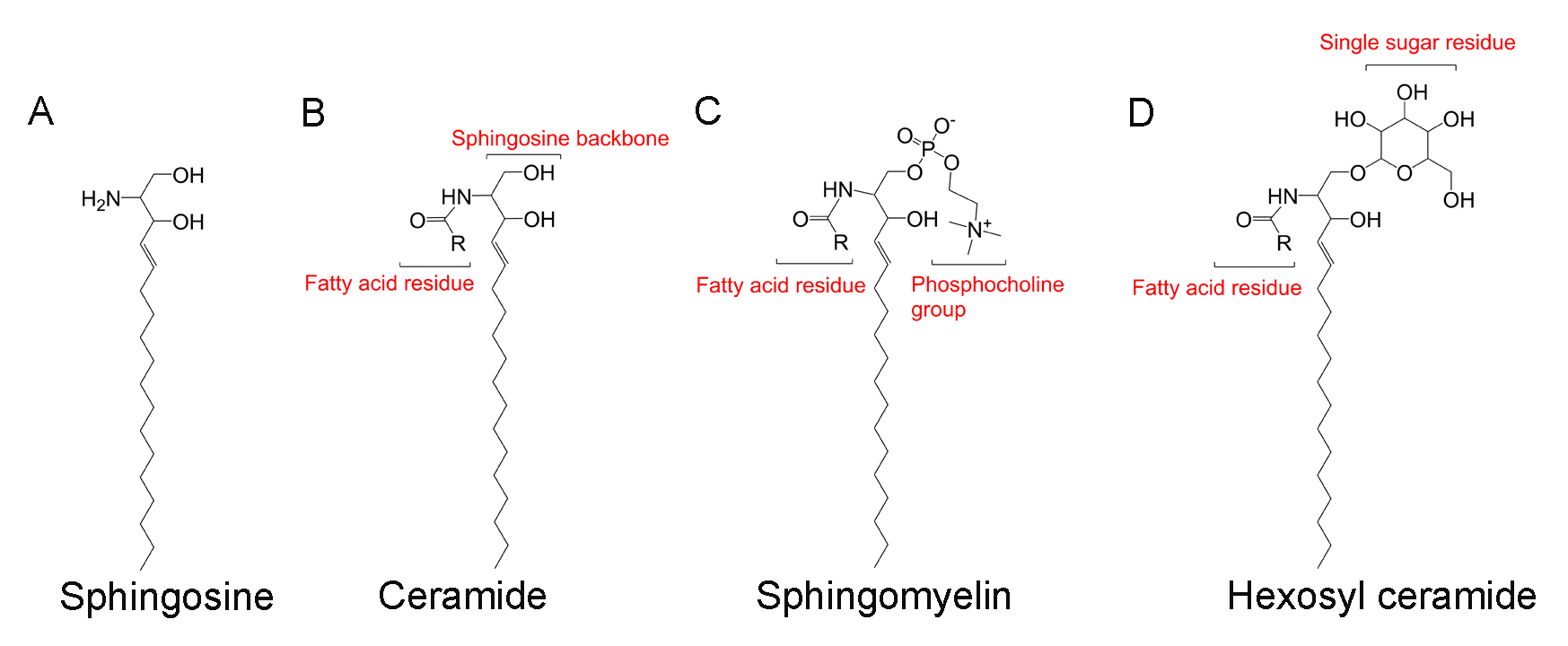

Supplement: Supplementary Figure 4 — The structural formula of sphinosine, ceramide, sphingomyelin and hexosyl ceramide drawn with ChemBioDraw Ultra 14.0. (A) Sphingosine with a C18 chain. (B) Ceramide, comprising a sphingosine backbone amide-linked to a fatty acid chain. (C) Sphingomyelin, formed by ceramide linking up with a phosphocholine head group. (D) Hexosyl ceramide, composed of a ceramide linked to a hexose. [file Image_4.TIF]
