# Supplementary material for: Structural and Lipidomic Alterations of Striatal Myelin in 16p11.2 Deletion Mouse Model of Autism Spectrum Disorder
Source: Front Cell Neurosci. 2021 Aug 12;15:718720. doi: 10.3389/fncel.2021.718720 (PMC8416256; doi:10.3389/fncel.2021.718720)
Supplement: Supplementary Table 1 — List of primers used for qRT-PCR. [file Table_1.docx]

**Table S1. List of primers used for qRT-PCR**

| **Gene Name** | **Sequences** |
| --- | --- |
| *Mag* | F: 5′-TGCTCACCAGCATCCTCACG-3′  R: 5′-AGCAGCCTCCTCTCAGATCC-3′ |
| *Mog* | F: 5′-CTGTTCTTGGACCCCTGGTT-3′  R: 5′-ACCTGCTGGGCTCTCCTT-3′ |
| *Mbp* | F: 5′-TACCTGGCCACAGCAAGTAC-3′  R: 5′-GTCACAATGTTCTTGAAG-3′ |
| *Plp1* | F: 5′-GTATAGGCAGTCTCTGCGCTGAT-3′  R: 5′-AAGTGGCAGCAATCATGAAGG |
| *Cnpase* | F: 5′-TGAGCTGGTCAGCTACTTTGG-3′  R: 5′-GATCTCTTCACCACCTCCTGC-3′ |
| *CerS2* | F: 5′-CCAGTCTTCTCAAAAAGTTCCG-3′  R: 5′-GGACCAGTAGAAGGAAAGTTCA-3′ |
| *Gapdh* | F: 5’-TGTGTCCGTCGTGGATCTGA-3’  R: 5’-CCTGCTTCACCACCTTCTTGA-3’ |
