# Supplementary material for: Structural and Lipidomic Alterations of Striatal Myelin in 16p11.2 Deletion Mouse Model of Autism Spectrum Disorder
Source: Front Cell Neurosci. 2021 Aug 12;15:718720. doi: 10.3389/fncel.2021.718720 (PMC8416256; doi:10.3389/fncel.2021.718720)
Supplement: Supplementary Table 2 — List of primary antibodies; List of secondary antibodies. [file Table_2.docx]

**Table S2.**

**List of primary antibodies**

| **Name** | **Source** | **Catalog Number** | **Dilution** | **Application** |
| --- | --- | --- | --- | --- |
| CERS2, Mouse | Santa Cruz | sc-390745 | 1:1000 | Western blot |
| GAPDH, Rabbit | ABclonal | A19056 | 1:3000 | Western blot |
| OLIG2, Mouse | Merck Millipore | MABN50 | 1:800 | Immunofluorescence (IF) |
| MYRF,  Rabbit | Prof. Mengsheng Qiu |  | 1:500 | IF |

**List of secondary antibodies**

| **Name** | **Source** | **Catalog Number** | **Dilution** | **Application** |
| --- | --- | --- | --- | --- |
| Goat anti-mouse IgG, HRP conjugate | Proteintech | SA00001-1 | 1:5000 | Western blot |
| Goat anti-rabbit IgG, HRP conjugate | Proteintech | SA00001-2 | 1:5000 | Western blot |
| Goat anti-Mouse IgG, Alexa Fluor Plus 488 conjugate | ThermoFisher | A32723 | 1:500 | IF |
| Goat anti-Rabbit IgG, Alexa Fluor Plus 488 conjugate | ThermoFisher | A32731 | 1:500 | IF |
